# COVID-19 and post-infectious myalgic encephalomyelitis/chronic fatigue syndrome: a narrative review

**DOI:** 10.1177/20499361211009385

**Published:** 2021-04-20

**Authors:** Sonia Poenaru, Sara J. Abdallah, Vicente Corrales-Medina, Juthaporn Cowan

**Affiliations:** Department of Medicine, The Ottawa Hospital, General Campus, 501 Smyth Road, Box 206, Ottawa, Ontario, K1H 8L6, Canada; Clinical Epidemiology Program, The Ottawa Hospital Research Institute, Ottawa, Ontario, Canada; Clinical Epidemiology Program, The Ottawa Hospital Research Institute, Ottawa, Ontario, Canada; Division of Infectious Diseases, Department of Medicine, University of Ottawa, Ottawa, Ontario, Canada; Clinical Epidemiology Program, The Ottawa Hospital Research Institute, Ottawa, Ontario, Canada; Division of Infectious Diseases, Department of Medicine, University of Ottawa, Ottawa, Ontario, Canada

**Keywords:** chronic fatigue syndrome, COVID-19, human coronavirus, myalgic encephalomyelitis, post-infectious fatigue, review

## Abstract

Coronavirus disease 2019 (COVID-19) is a viral infection which can cause a variety of respiratory, gastrointestinal, and vascular symptoms. The acute illness phase generally lasts no more than 2–3 weeks. However, there is increasing evidence that a proportion of COVID-19 patients experience a prolonged convalescence and continue to have symptoms lasting several months after the initial infection. A variety of chronic symptoms have been reported including fatigue, dyspnea, myalgia, exercise intolerance, sleep disturbances, difficulty concentrating, anxiety, fever, headache, malaise, and vertigo. These symptoms are similar to those seen in myalgic encephalomyelitis/chronic fatigue syndrome (ME/CFS), a chronic multi-system illness characterized by profound fatigue, sleep disturbances, neurocognitive changes, orthostatic intolerance, and post-exertional malaise. ME/CFS symptoms are exacerbated by exercise or stress and occur in the absence of any significant clinical or laboratory findings. The pathology of ME/CFS is not known: it is thought to be multifactorial, resulting from the dysregulation of multiple systems in response to a particular trigger. Although not exclusively considered a post-infectious entity, ME/CFS has been associated with several infectious agents including Epstein–Barr Virus, Q fever, influenza, and other coronaviruses. There are important similarities between post-acute COVID-19 symptoms and ME/CFS. However, there is currently insufficient evidence to establish COVID-19 as an infectious trigger for ME/CFS. Further research is required to determine the natural history of this condition, as well as to define risk factors, prevalence, and possible interventional strategies.

## Introduction

Coronavirus disease 2019 (COVID-19) is a viral illness caused by the severe acute respiratory syndrome coronavirus 2 (SARS-CoV-2).^1^ The acute illness phase has been well characterized: symptoms can include a variety of respiratory, neurologic, gastrointestinal, and vascular manifestations that generally last no more than 2–3 weeks.^2^ However, some COVID-19 patients experience a prolonged convalescence phase and continue to have symptoms for several months after the initial infection.^1^ Data from narrative patient experiences after COVID-19 infection and early observational studies suggest a syndrome similar to myalgic encephalomyelitis/chronic fatigue syndrome (ME/CFS), a chronic multi-system illness that has been associated with other infections.^3,4^ The term “long COVID” has been used to describe this entity by many researchers. However, an agreed-upon case definition does not yet exist.^5^ In this article, we will use “post-acute COVID-19 symptoms” to describe symptoms lasting longer than 3 weeks. We aim to review similarities and differences between ME/CFS and post-acute COVID-19 symptoms, potential mechanisms of pathogenesis, and management strategies.

## ME/CFS

### Epidemiology and diagnostic criteria

ME/CFS is a complex chronic multi-system illness associated with a variety of constitutional and neurocognitive symptoms. It has a prevalence of 0.17–0.89% in the general population and occurs more frequently in women.^6^ Many other predisposing factors such as age, pre-existing psychiatric conditions, socio-economic status, and activity level have been inconsistently associated with increased risk of developing ME/CFS.^7^ Although the pathogenesis is not well understood, many cases of ME/CFS are thought to be triggered by infection.^8^ For example, a large retrospective study of 837 patients found symptoms of acute infection (fever, upper respiratory tract infection, flu-like illness, or gastroenteritis) preceded ME/CFS symptom onset in 77% of patients.^9^ This is in agreement with rates of preceding infectious symptoms seen in other studies.^10,11^ However, the true extent to which infections contribute to the development of ME/CFS remains undefined.^8,12,13^ A higher rate of stressful life events has also been observed in the 3 months prior to onset of ME/CFS, while physical stressors such as severe injury or surgery have not been associated with ME/CFS.^10,11^ In many cases no particular trigger can be identified.^8,10^

The characterization of ME/CFS remains controversial. The pathogenesis of the disease is poorly understood and there are no specific diagnostic physical signs or biomarkers. As a result there is no universally agreed-upon definition of ME/CFS. Twenty-five different diagnostic criteria have been proposed so far.^8,12,14–18^

The ME/CFS research community has commonly used the revised Center for Disease Control (CDC) criteria defined by Fukuda *et al*. in 1994.^8,16,17^ This case definition has been criticized as being non-specific by focusing only on fatigue as the key symptom. Epidemiologic studies have found up to a five-fold higher prevalence of ME/CFS using these CDC criteria compared with the more recent and stringent International Consensus Criteria (ICC) or Canadian Consensus Criteria (CCC), as summarized in Table 1.^7,15–19^

**Table 1.** Three commonly used diagnostic criteria for ME/CFS.

1994 CDC Criteria (Fukuda *et al*.)^16^	2006 Canadian Consensus Criteria^19^	2015 Institute of Medicine Criteria^13^
Required symptoms:	Required symptoms:	Required symptoms:
Persistent or relapsing chronic fatigue	Persistent or relapsing chronic fatigue	Persistent or relapsing chronic fatigue
Lasting 6 months	Lasting 6 months	Lasting 6 months
New or definite onset	New or definite onset	New or definite onset
Not due to ongoing exertion	Not due to ongoing exertion	Not due to ongoing exertion
Not alleviated by rest	Substantial reduction in daily activities	Not alleviated by rest
Substantial reduction in daily activities	Not due to other medical condition	Substantial reduction in daily activities
Not due to other medical condition	Post-exertional malaise or fatigue	Not due to other medical condition
Additional symptoms:	Worsening symptoms after exertion	Post-exertional malaise or fatigue
(Four or more concurrently present)	Inappropriately low physical and mental stamina	Worsening symptoms after exertion
Impaired concentration or memory	Pathologically slow recovery >24 h	Mentally and physically drained following minimal exertion
Sore throat	Sleep dysfunction	Failure to reproduce results on exercise tests 24 h apart
Tender cervical/axillary lymphadenopathy	Unrefreshing sleep	Sleep dysfunction
Muscle pain	Circadian rhythm disturbance	Unrefreshing sleep
Pain in several joints	Pain – widespread or migratory	Circadian rhythm disturbance
New headaches	Headaches, joint, or muscle pain	Additional Symptoms:
Unrefreshing sleep	Neurologic or cognitive manifestations	(One symptom category)
Malaise after exertion	Confusion or disorientation	Cognitive impairment
	Impaired concentration or memory	Confusion or disorientation
	Information processing difficulty	Impaired concentration or memory
	Perceptual or sensory disturbances	Information processing difficulty
	Ataxia, weakness, or fasciculation	Altered executive function/attention
	Overload phenomena (sensory or emotional)	Impaired psychomotor function
	Additional symptoms:	Orthostatic intolerance
	(One symptom from two or more categories)	Light-headedness, imbalance, or fainting with postural change
	Autonomic manifestations	Delayed postural symptoms
	Orthostatic intolerance	Abnormal blood pressure/tachycardia on postural testing
	Irritable bowel or bladder dysfunction	
	Palpitations or exertional dyspnea	
	Neuroendocrine manifestations	
	Loss of thermostatic ability	
	Intolerance of extremes of temperature	
	Immune manifestations	
	Tender lymph nodes	
	Sore throat/flu-like symptoms	

The most recent Institute of Medicine diagnostic criteria published in 2015 characterize ME/CFS as a spectrum of five core symptoms: fatigue, post-exertional malaise, cognitive changes (impaired memory, concentration, information processing), sleep disturbance (unrefreshing sleep, circadian rhythm reversal), and orthostatic intolerance.^13^ Post-exertional malaise in particular is considered an important feature of ME/CFS that distinguishes it from other chronic illnesses such as fibromyalgia, somatic depression, or primary sleep disorders.^7,18^ A wide variety of secondary symptoms such as pain, sensorimotor abnormalities, arthralgias, gastrointestinal symptoms (nausea, bloating, irritable bowel), urinary symptoms (frequency, urgency), sore throat, and lymphadenopathy (cervical and/or axillary) are included in some criteria but not required for diagnosis.^8,13–16,20,21^ Symptoms must not be relieved by rest and must persist for more than 6 months in the absence of any significant clinical or laboratory findings.^8,13^

### Post-infectious ME/CFS

Clusters of illnesses resembling ME/CFS have been observed throughout the 20th century following institutional or epidemic infectious outbreaks.^12,22–25^ Symptom patterns following these outbreaks include chronic fatigue, lethargy, malaise, sleep disturbance, and poor concentration, often exacerbated by physical exertion or stress.^23,25^ Although diagnostic criteria did not exist at the time, this spectrum of symptoms is highly suggestive of post-infectious ME/CFS.^25,26^ As both ME/CFS case definitions and diagnostic methods in microbiology evolved over time, a clearer link between infection and ME/CFS has emerged.

Infectious mononucleosis caused by the Epstein–Barr Virus (EBV) is the infection most consistently associated with the development of ME/CFS.^13^ A prospective study of 301 adolescents diagnosed with acute EBV infection by positive Monospot found that 13% of participants met 1994 CDC criteria for ME/CFS 6 months later, and 4% had still not recovered after 24 months.^27^ This is in agreement with previous reports of EBV-associated chronic fatigue in adults.^28,29^ Similar rates of post-infectious fatigue were reported following Q Fever or Ross River Virus infection (12% at 6 months by 1994 CDC criteria), and about 20% following West Nile Virus infection and other glandular fevers.^23,28,30,31^

There is evidence suggesting that a wider array of viral and bacterial illness can also be associated with increased risk of developing ME/CFS.^28,32^ For example, in a prospective cohort study of 618 patients diagnosed with a non-specific viral infection by their primary care provider 12.9% met criteria for chronic fatigue (using an independently validated fatigue scale) at 6 months.^32^ In a longitudinal study following patients with acute EBV, Ross River Virus, Q fever, or serologically unconfirmed febrile illness, the prevalence and severity of chronic fatigue, functional impairment, and neurocognitive disturbance post-infection was the same regardless of specific infectious trigger.^28^

### ME/CFS and viral epidemics

Following the 1918 influenza pandemic, up to 40% of survivors remained chronically unwell with a variety of symptoms including fatigue, lethargy, and difficulty concentrating which were exacerbated by physical exertion.^23,25^ More recently, a population health registry surveillance study in Norway identified an increased incidence of ME/CFS diagnosis after the 2009 H1N1 pandemic.^33^ Survivors of recent coronavirus outbreaks, including severe acute respiratory syndrome (SARS) in 2002 and Middle East respiratory syndrome (MERS) in 2012, reported multiple persistent symptoms including fatigue, widespread pain, unrefreshing sleep, post-exertional malaise, and changes to cognition.^34–38^ One study of 233 SARS survivors found that 27.1% met criteria for ME/CFS (as defined by 1994 CDC criteria) at 41 months post-infection.^36^ A meta-analysis of post-infectious symptoms in MERS and SARS found that 19.3% of patients experienced ongoing fatigue up to 39 months after infection.^35^

In addition to persistent fatigue, psychiatric and neurocognitive complications following influenza and coronavirus epidemics have been observed.^22,31,39^ For example, first-time hospitalizations for psychiatric disorders increased by a factor of 7.2 for several years after the 1918 pandemic.^22^ More recently, a study of 37 patients with H1N1 influenza acute respiratory distress syndrome found high rates of anxiety (50%), depression (28%), and post-traumatic stress disorder (PTSD) (41%) after 1 year.^40^ Survivors of H7N9 influenza reported persistently reduced mental health scores on 36-item short form survey after 24 months.^41^ A meta-analysis of long-term symptoms in SARS and MERS survivors found a high prevalence of depression (14.9%), anxiety (14.8%), and PTSD (32.2%) compared with population rates of approximately 7%.^35,42,43^ One study found that the prevalence of comorbid psychiatric conditions was significantly higher in patients with post-SARS ME/CFS, but found no association with initial illness severity, other medical comorbidities, age, or gender.^36^ In contrast, pre-existing psychiatric conditions are not consistently associated with EBV-associated ME/CFS.^31^ The higher rates of both ME/CFS and psychiatric diagnoses observed in SARS survivors may reflect the role of stressful life events as an independent risk factors for developing ME/CFS.^10,11,36^

The existing evidence suggests a temporal relationship between viral epidemics and chronic post-infectious symptoms that are consistent with the criteria for ME/CFS.

#### Proposed mechanisms of post-infectious ME/CFS

**Figure 1.**
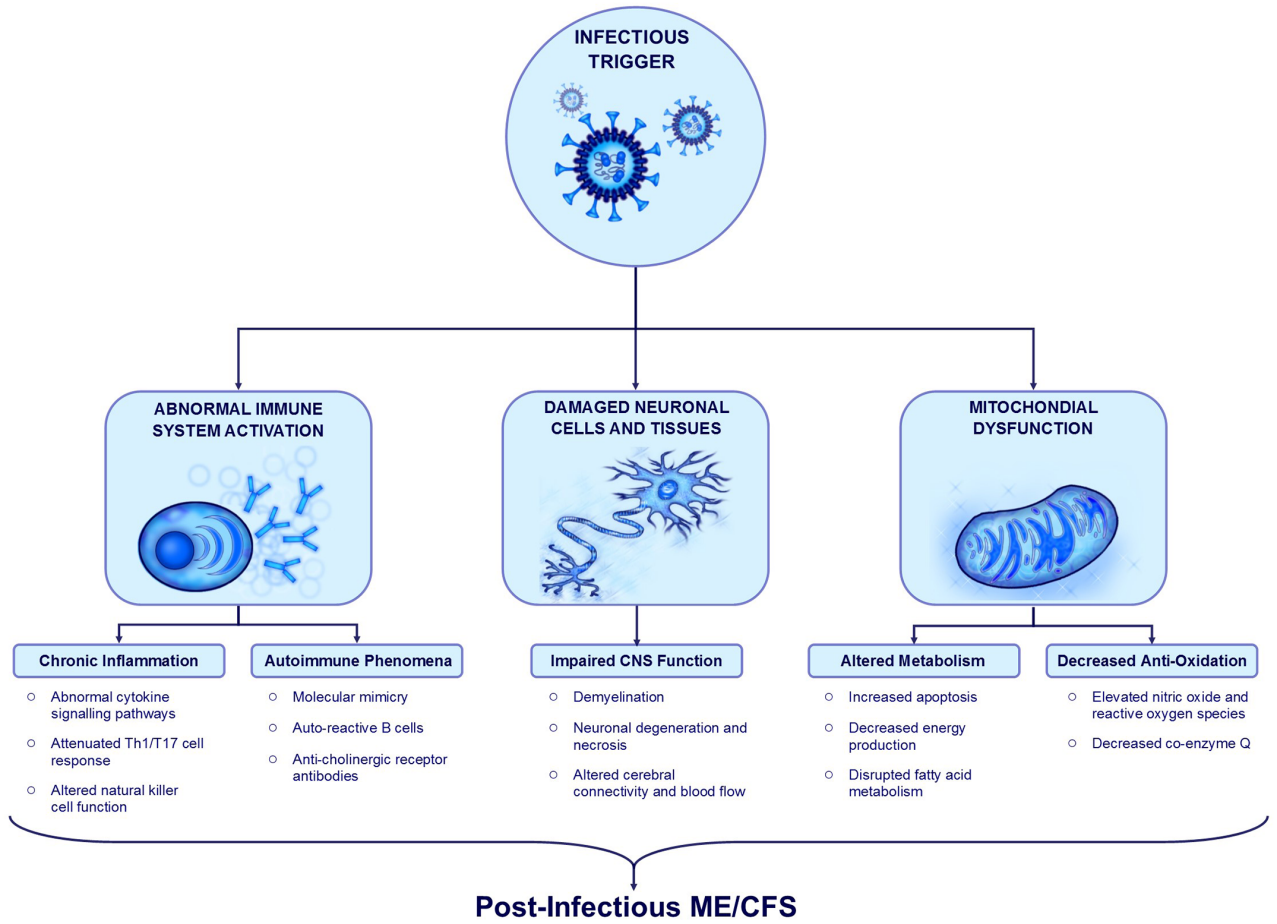
Summary of post-infectious ME/CFS mechanisms. Infective agents activate and alter immune system function leading to chronic inflammation, increased pro-inflammatory cytokine signaling, and abnormal function of multiple cell types including Th1, Th17, T-regulatory, and natural killer cells. Autoimmune mechanisms such as molecular mimicry and auto-reactive bystander cell activation can also be triggered during acute infection. Infective agents with neuro-invasive potential can cause inflammatory and ischemic damage to central nervous system cells and tissues, resulting in neuronal degeneration, demyelination, and subsequent functional impairment. Infective agents may also cause structural damage to mitochondria, leading to decreased energy production, altered metabolism, and reduced anti-oxidant function. These processes may underlie the symptoms reported in post-infectious ME/CFS.

Although post-infectious ME/CFS has been associated with a variety of different pathogens, the incidence and disease manifestations are similar regardless of inciting pathogen.^28,32,44^ Symptoms persist long after clearance of the initial infection and occur in the absence of any significant abnormality detectable with diagnostic testing.^8,13^ This has resulted in a “hit-and-run” hypothesis, which suggests that susceptible individuals experience persistent dysregulation of immune, neurologic, and metabolic pathways following exposure to an infectious trigger (Figure 1).^44^ Multiple organ systems and signaling pathways have been investigated in both human and animal models. However, findings are not consistent between studies. The mechanism of post-infectious ME/CFS remains poorly understood, and is likely multifactorial.

### Immune/inflammatory mechanisms

Post-infectious ME/CFS has often been purported as an inflammatory disorder in which an infectious pathogen triggers an abnormal systemic immune response that persists beyond clearance of the infection.^8,44^ Proposed mechanisms linking acute infection and chronic immune system dysregulation in the pathogenesis of ME/CFS include altered immune cell function, abnormal signaling pathways, chronic inflammation, and autoimmune phenomena.^44–47^

Lasting patterns of altered immune system function favoring a pro-inflammatory milieu following an acute infection have been demonstrated in animal models.^45,46,48^ For example, in mice, the infection of astrocytes and microglia with a murine coronavirus (MHV-A59) creates a persistent pro-inflammatory environment within the central nervous system (CNS) which is not seen following exposure to non-neurotropic virus or in uninfected controls.^46^ Increased levels of five pro-inflammatory cytokines (interleukin-12 p40, interleukin-6, interleukin-15, interleukin-1, and tumor necrosis factor-α) were detected in the brain during the acute infectious encephalitis phase and remained persistently elevated within the spinal cord at 30 days post-infection.^46^

Findings suggestive of systemic chronic inflammation and abnormal pro-inflammatory cytokine expression have also been observed in ME/CFS patients.^49^ For examples, many studies have suggested that dysregulation of cytokine networks may play a role in ME/CFS, including a recent meta-analysis which found altered levels of tumor necrosis factor α, transforming growth factor β, interleukin-2, and interleukin-4 compared with healthy controls.^50^ However, important differences in cytokine levels are highly inconsistent between studies: a separate meta-analysis analyzing 64 cytokines found no significant association with ME/CFS.^51^ There is some emerging evidence that cytokine signaling and network connections are more significant than circulating cytokine levels alone.^47^

Abnormal immune cell function may also be associated with ME/CFS. For example, an attenuated TH1/TH17 cell response has been described in ME/CFS, which is similar to the pattern seen in latent infections such as EBV.^25,47^ Altered natural killer and T-regulatory cell function has also been reported in ME/CFS.^52^ There may be a genetic basis for predisposition to chronic immune system dysregulation after an infectious trigger.^53,54^ However, there is no inflammatory biomarker, altered cellular function, or genetic polymorphism that is seen consistently across cases of ME/CFS.^47,50,51^

Tissue damage sustained during acute infection leading to activation of auto-reactive bystander cells and molecular mimicry have been proposed as potential autoimmune mechanisms.^44,49^ For example, in severe COVID-19 infections significantly increased levels of anti-nuclear antibodies and rheumatic factor have been detected, suggesting heightened activation of auto-reactive B cells.^55^ Antibodies against muscarinic and adrenergic receptors have been identified in ME/CFS and are thought to be associated with postural orthostatic symptoms.^52^ However, no specific B-cell phenotype or auto-antibody has been consistently linked with ME/CFS.^56^

In summary, the immune system appears to be impacted in post-infectious ME/CFS. However, the precise mechanism is unclear and likely involves multiple pathways.

### Central nervous system involvement

Several core symptoms of ME/CFS (impaired cognition, sleep disturbance) as well as some secondary symptoms (sensory overload phenomena, motor symptoms) may reflect altered CNS function.

A meta-analysis of imaging findings in ME/CFS found a greater proportion of altered cerebral blood flow, structural cortical abnormalities, focal inflammation, and changes to functional connectivity compared with healthy controls.^20^ It is not clear whether any specific neurocognitive deficits occur as a result of these structural abnormalities. Likewise, the mechanism of these changes as they relate to infectious triggers is not known.

Many viruses, including some coronaviruses, are known to have neuro-invasive potential and can cause inflammatory damage to CNS tissue.^57^ For example, SARS-CoV1 isolated from human brain tissue and cerebrospinal fluid has been associated with edema, neuronal degeneration, demyelination, and necrosis in severe cases.^45,48,58,59^ Increased risk of cerebral ischemic and microvascular events has been reported in acute SARS, MERS, and COVID-19 infection.^60,61^ There is some evidence of a functional association between certain viral infections and chronic neurologic disease. For example, one specific strain of human coronavirus (HCoV-0C43) that is known to cause a febrile respiratory and gastrointestinal illness in humans was found to be significantly more prevalent in the CNS tissue of people with multiple sclerosis than in healthy controls.^58^ A sleep study in SARS survivors who were unable to return to work due to chronic symptoms found a high proportion of rapid eye movement and alpha electroencephalographic sleep anomalies commonly seen in ME/CFS patients, suggesting a common pathologic mechanism.^37^

Although a multitude of post-infectious changes to inflammatory, autoimmune, and cellular signaling mechanisms within the CNS have been identified, the role of each of these in the pathogenesis of post-infectious ME/CFS remains unclear.^20,44,49,60^ A causal relationship between acute infection, altered CNS structure and function, and post-infectious ME/CFS symptoms has not been clearly established.

### Mitochondrial function and fatigue

Fatigue is a defining feature in both ME/CFS and primary mitochondrial disorders, which has led to a large body of research investigating the connection between mitochondrial function and ME/CFS.^62^

Alterations in mitochondrial structure, metabolism, and energy production within muscle tissues may be associated with the fatigue and post-exertional malaise seen in ME/CFS.^44,62^ One study examining muscle biopsy samples in a population of 50 people diagnosed with post-viral fatigue syndrome (by Holmes 1988 criteria) found mitochondrial degeneration, pleomorphic features, and significant structural abnormalities in 80% of cases, as compared with minor structural changes seen in only 52% of healthy controls.^63^

Mitochondrial enzymes involved in inflammatory and anti-oxidant pathways are of particular interest as drivers of orthostatic intolerance and post-exertional malaise due to their involvement in peripheral vasodilation and autonomic regulation of the cardiovascular system.^64–67^ A small prospective study of gene expression in five people with post-EBV ME/CFS found significant differences in several genes associated with mitochondrial fatty acid metabolism, oxidation, membrane function and apoptosis relative to Human Leukocyte Antigen-matched healthy controls.^68^ These findings are in keeping with other mitochondrial function studies in ME/CFS which have found alterations in enzyme levels associated with oxidation (nitrous oxide, radical oxygen species), fatty acid metabolism, and energy production.^65,69^ One of the most commonly studied is the anti-oxidant coenzyme Q10, which has been found to be lower in ME/CFS than in healthy controls in some studies.^62,64^

There is currently insufficient data to classify ME/CFS as a mitochondrial disorder or to link post-infectious ME/CFS with mitochondrial dysfunction. Most studies are either limited by small sample size, difficult to compare based on different diagnostic criteria and case definitions, or inconsistent in their results. A clear plausible pathway to explain lasting mitochondrial abnormalities after acute infections is also lacking: two systematic reviews on the role of mitochondrial function in ME/CFS found no significant agreement in structural, genetic, metabolic, or oxidative pathway abnormalities between studies.^62,70^

## COVID-19 and ME/CFS

Observational studies have described persistent symptoms of acute COVID-19 as lasting at least 3 weeks from disease onset, with some patients reporting lingering symptoms for longer than 4 months.^2,22,71–78^ A variety of chronic symptoms, including fatigue, dyspnea, joint pain, myalgia, sleep disturbances, difficulty concentrating, memory problems, cough, anosmia, anxiety, headache, fever, and vertigo have been reported.^74–78^ Many narrative reports of post-acute COVID-19 patient experiences describe profound fatigue and cognitive changes that are exacerbated by physical activity or stress.^3,79–81^ Although these symptoms parallel those that are seen in post-infectious ME/CFS, data supporting COVID-19 as an infectious trigger for ME/CFS are limited.

The exact prevalence and expected duration of post-acute COVID-19 symptoms is under ongoing investigation. Some studies have reported at least one persistent symptom in 75% of post-COVID patients at follow-up ranging from 7 to 12 weeks later.^82,83^ A recent systematic review of 28 post-COVID-19 symptom studies found that fatigue, dyspnea, and anosmia were the most frequently reported symptoms lasting more than 3 weeks.^84^ However symptom duration, patient populations, and length of follow-up are highly variable between studies, with reported rates of full recovery between 13% and 86% at follow-up ranging from 30 to 186 days (Table 2).^71–73,75,78,83,85–103^

**Table 2.** Post-acute COVID-19 symptom frequency.

Author	Country	Population	Sample size	Follow-up (days)	Recovered at follow-p (%)	Most common symptoms (%)	Risk factors
Huang *et al.*^71^	China	Inpatient	1733	186	24	Fatigue/myalgia (63)Sleep disturbance (26)Hair loss (22)	AgeFemale sexDisease severity
Nehme *et al.*^89^	Switzerland	Inpatient	669	43	68	Fatigue (–)Dyspnea (–)Anosmia (–)	–
Khalaf *et al.*^90^	Egypt	Mixed (51% Inpatient, 49% Outpatient)	538	82	15	Fatigue (59)Subjective fever (47)Diarrhea (24)	Disease severityHydroxychloroquine useAzithromycin useMultivitamin use
Xiong *et al.*^86^	China	Inpatient	538	97	50	Fatigue (28)Diaphoresis (24)Post-exertion polypnea (21)	AgeFemale sexHospital length of stay
Chopra *et al.*^91^	United States	Inpatient	488	60	–	Physical limitation (39)Exertional dyspnea (23)Anosmia (13)	–
Mohamed-Hussain *et al.*^92^	Egypt	Mixed (76% Inpatient, 24% Outpatient)	444	35	20	–	AgeFemale sexDisease severitySeasonal flu vaccineSmoking historyAny medical comorbidity
Galal *et al.*^93^	Egypt	Mixed (24% Inpatient, 76% Outpatient)	430	–	14	Myalgia (60)Arthralgia (57)Physical limitation (57)	Any medical comorbidityDisease severityInfluenza vaccination
Mandal *et al.*^83^	England	Inpatient	384	54	28	Fatigue (67)Dypsnea (53)Cough (34)Sleep disturbance (61)Dyspnea (55)	–
Moradian *et al.*^94^	Iran	Inpatient	200	42	42	Dyspnea (20)Weakness (19)Myalgia (18)	–
Jacobs *et al.*^87^	United States	Inpatient	183	35	27	Fatigue (55)Myalgia (51)Dyspnea (45)	AgeFemale sex
Petersen *et al.*^95^	Faroe Islands	Outpatient	180	125	47	Fatigue (–)Anosmia (–)Myalgia (–)	Age
Pilotto *et al.*^88^	Italy	Inpatient	165	97	50	Fatigue (34)Memory loss (31)Sleep disturbance (30)	AgeDisease severity
Townsend *et al.*^73^	Ireland	Mixed (48% inpatient, 52% outpatient)	153	75	38	Fatigue (48)	–
Carfi *et al.*^72^	Italy	Inpatient	143	60	13	Fatigue (53)Dyspnea (43)Arthralgia (27)	–
Galvan-Tejada *et al.*^96^	Mexico	Inpatient	141	36	16	Cough (25)Anosmia (24)Emesis (15)	–
Wang *et al.*^78^	China	Inpatient	131	30	86	Cough (9)Dyspnea (2)Pharyngitis (2)	–
Garrigues *et al.*^97^	France	Inpatient	120	111	–	Fatigue (55)Dyspnea (42)Memory loss (34)	–
Pellaud *et al.*^85^	Switzerland	Inpatient	116	30	37	Fatigue (67)Respiratory symptoms (56)Anosmia (10)	–
Varghese *et al.*^98^	Germany	Mixed (9% Inpatient, 91% Outpatient)	116	66	79	Fatigue (11)Dyspnea (6)Anosmia (5)	Reduced serum IgA
Arnold *et al*.^82^	England	Inpatient	110	90	26	Fatigue (39)Dyspnea (39)Insomnia (24)	–
Halpin *et al.*^75^	England	Inpatient	100	48	–	Fatigue (63)Dyspnea (50)Post-traumatic stress disorder symptom (31)	–
Darley *et al.*^99^	Australia	Mixed (88% Inpatient, 12% Outpatient)	78	69	60	Fatigue (22)Dyspnea (19)Chest tightness (5)	–
Wong *et al.*^100^	Canada	Inpatient	78	90	24	Reduced quality of life (51)Dyspnea (50)Cough (23)	–
Stavem *et al.*^101^	Norway	Outpatient	70	117	58	Dyspnea (16)Anosmia (12)Dysgaeusia (10)	Medical comorbiditiesNumber of acute symptoms
Miyazato *et al.*^102^	Japan	Inpatient	63	120	–	Dyspnea (11)Fatigue (10)Anosmia (10)	–
Zhao *et al.*^103^	China	Inpatient	55	64–93	–	Gastrointestinal (31)Headache (18)Fatigue (16)	–

An observational study investigating post-acute COVID-19 symptoms as defined by ME/CFS criteria does not yet exist. However, the high prevalence of persistent fatigue is very relevant to ME/CFS. A large prospective cohort study of 1733 patients admitted to hospital with COVID-19 found that 63% of them were still experiencing fatigue/myalgia at 6 months post-discharge.^71^ However, the presence of chronic fatigue alone is insufficient to diagnose ME/CFS. Future studies investigating other key features of ME/CFS, such as post-exertional malaise and neurocognitive changes, will be required to establish a diagnosis.

As seen in previous coronavirus outbreaks, dyspnea is the other most common persistent symptom, reported in up to 56% of inpatients at follow-up ranging from 1 to 6 months.^71,75,85^ Dyspnea and exercise intolerance in the context of ME/CSF are mainly recognized as having a strong orthostatic component, a feature not clearly described in post-COVID-19 cases. Certain abnormalities on pulmonary testing have also been detected in this population: for example, studies in post-COVID-19 patients have demonstrated mild restrictive spirometry and imaging abnormalities in more than half of patients. However, these findings do not correlate well with initial disease severity or overall symptom burden.^74,76,77^ It is not clear in these studies whether dyspnea out of proportion to physical findings occurs in conjunction with postural symptoms such as tachycardia or hypotension, which would suggest an orthostatic component more in keeping with ME/CFS, or secondary to other factors such as deconditioning or post-viral lung injury.

An association between chronic symptoms and age, illness severity, and female gender was seen in some studies.^71,86–88^ Other proposed risk factors, including ethnicity, psychiatric condition, number of medical comorbidities, or obesity were not consistently associated with post-acute COVID19 symptoms.^72,73,75,77,82^ As seen in ME/CFS, there was no biomarker (complete blood count, lymphocyte count, neutrophil count, monocyte count, D-dimer, C-reactive protein, lactate dehydrogenase, interleukin-6, CD-25, liver function tests, or creatinine) differentiating patients who remained symptomatic from those who returned to baseline health.^73,82,83^

## Management options

The approach to treating ME/CFS generally focuses on symptom management and minimizing unnecessary investigations.^2^ However, a thorough workup to rule out other organic cause for ME/CFS symptoms must be done prior to giving this diagnosis. In post-acute COVID-19 this includes outpatient pulmonary imaging for people with severe respiratory disease during acute illness, as well as screening and concurrent management for comorbid psychiatric illness.^2,22,104^

The National Institute for Health and Care Excellence guidelines on ME/CFS currently recommends graded exercise therapy and cognitive behavioral therapy.^77^ However, more recent evidence suggests that graded exercise therapy may accentuate post-exertional malaise in some patients.^105–107^ This effect has been demonstrated in patient narratives of post-acute COVID-19 symptoms, who describe even minimal physical exertion as exacerbating their symptoms and rendering them bedbound for several days.^3,79–81^ For this reason, some experts have cautioned against graded exercise therapy on the management of fatigue in post-acute COVID-19.^108^

Recent expert opinions on the management of post-acute COVID-19 in primary care recommend an approach based on conservative symptom relief strategies, referral to specialists for co-management of comorbidities, and a multidisciplinary approach to social, cultural, and financial support.^2^ However, further research will be required to determine the benefit of any specific treatment for this condition.

## Discussion

The evidence for post-infectious ME/CFS following COVID-19 is not as strong as for other viruses such as EBV. Although persistent fatigue has been described extensively in post-acute COVID-19 symptom studies, no study has used ME/CSF criteria to characterize chronic fatigue in conjunction with other key symptoms and common disease manifestations.^5,71,82,83^ Another limitation is the degree of variability among different ME/CSF diagnostic criteria. Most post-infectious ME/CFS studies continue to use the 1994 CDC diagnostic criteria, which do not require the presence of other hallmark features of ME/CFS such as post-exertional malaise, cognitive changes, sleep disturbances, or orthostatic intolerance for diagnosis.^7,8,16–19^ This leads to difficulty interpreting the significance of individual chronic symptoms within the context of a post-infectious ME/CFS diagnosis. Diagnosis of post-infectious ME/CFS in COVID-19 patients is further limited by its emerging infection status, as a duration of follow-up of at least 6 months is required to make this diagnosis.

Some symptoms seen in post-acute COVID-19 may occur as a consequence of critical illness or as a side effect of treatments such as steroids. For example, dyspnea is seen in up to 36% of people diagnosed with ME/CFS and is considered part of the broader category of orthostatic intolerance, along with postural tachycardia and hypotension.^109^ However, the dyspnea reported in post-COVID studies is not clearly described as a manifestation of orthostatic intolerance and may in fact represent fibrosis following inflammatory lung injury.^76–78^ This theory would be supported by the presence of clinically detectable abnormalities on imaging and pulmonary function testing in post-acute COVID-19 patients.^73,74^ Similar findings can be seen in survivors of acute respiratory distress syndrome, suggesting an organic cause for dyspnea.^110–112^ Other complications of critical illness and acute respiratory distress syndrome such as loss of muscle mass, deconditioning, steroid-induced myopathy, and multi-organ failure are correlated with poorer long-term health outcomes, chronic fatigue, and decreased functional capacity.^111^ There is some overlap between these outcomes and symptoms of ME/CFS. However, it is important to note that multiple post-acute COVID-19 studies have found no association between illness severity, presence of chronic symptoms, and objective measures of respiratory function, suggesting an alternate mechanism of pathogenesis.^72,73,75,77,82^

The importance of the higher rate of psychiatric comorbidities seen following epidemic outbreaks is similarly unclear. This association is likely caused by external stressors rather than due to the infection itself.^11,35,42^ While specific psychiatric conditions have not been consistently associated with increased risk of post-infectious ME/CFS, other psychosocial factors such as stressful life events, persistent high levels of anxiety, and reduced community support may play a role.^7,17,28,29^ Evidence from prior viral epidemics suggests that this period of multiple stressful life events may be an independent risk factor for developing ME/CFS; it will be difficult to separate the impact of pandemic-associated stress from the impact of the infection itself in defining COVID-19 as a risk factor for ME/CFS.

Although the symptom patterns seen in post-acute COVID-19 are similar to those seen in ME/CFS, further investigation with longer periods of follow-up and clearly defined diagnostic criteria will be required to establish COVID-19 as an infectious trigger for ME/CFS.

## Bottom line

- Many post-acute COVID-19 symptoms resemble post-infectious ME/CFS
- Acute disease severity does not clearly correlate with persistent symptoms
- Long-term monitoring of post-acute COVID-19 symptoms and screening for common comorbid conditions is essential
- Further research is required to establish COVID-19 as an infectious trigger for ME/CFS as well as to define risk factors, prevalence, natural history, and possible interventional strategies to treat this condition
